# Overexpressing PLOD family genes predict poor prognosis in gastric cancer

**DOI:** 10.7150/jca.35763

**Published:** 2020-01-01

**Authors:** Shan-Shan Li, Yi-Fan Lian, Yan-Lin Huang, Yue-Hua Huang, Jian Xiao

**Affiliations:** 1 Department of Medical Oncology, the Sixth Affiliated Hospital of Sun Yat-sen University, Guangzhou, China; 2 Guangdong Provincial Key Laboratory of Liver Disease Research, the Third Affiliated Hospital of Sun Yat-sen University, Guangzhou, China; 3 Department of Infectious Diseases, the Third Affiliated Hospital of Sun Yat-sen University, Guangzhou, China

**Keywords:** PLOD, gastric cancer, prognosis, bioinformatics

## Abstract

Procollagen-lysine, 2-oxoglutarate 5-dioxygenases (PLODs) are a set of enzymes involved in the hydroxylation of lysine and stabilization of collagen by crosslinks. Previous studies have highlighted that overexpressing PLOD genes were related to the progression, migration and progression of different human cancers. However, the diverse expression patterns and prognostic values of PLOD genes remain to be elucidated in gastric cancer (GC). In this study, we mined the expression and survival data in GC patients through ONCOMINE, UALCAN and Kaplan-Meier Plotter database. STRING portal couple with DAVID was used to establish a functional protein interaction network of PLOD family genes and analyze the GO and KEGG enriched pathways. Differential gene expression correlated with PLOD family genes was identified with LinkedOmics. We found that PLOD1, 2 and 3 were up-regulated in GC patients compared with normal tissues. High expression levels of PLOD1 and PLOD3 were associated with shorter overall survival (OS), first progression (FP) and post progression survival (PPS) while high expression level of PLOD2 was only associated with shorter FP in all GC patients. Specifically, only high PLOD2 expression had significant correlation with shorter OS, FP and PPS in the diffuse type GC patients. Furthermore, combinatorial use of expressions of all PLOD genes was a superior prognostic indicator for GC patients. Pathway analysis confirmed that PLOD family genes mainly participate in regulating the collagen metabolism and extracellular matrix constitution, and the cellular adaptor protein SHC1, which helps to transduce an extracellular signal into an intracellular signal, could be the regulatory module mediating PLOD's effect on GC. Therefore, we propose that individual PLOD genes or PLOD family genes as a whole could be potential prognostic biomarkers for GC.

## Introduction

As a major component of tumor microenvironment, extracellular matrix (ECM) plays an important role in cancer development and progression, which consists of structural proteins, glycoproteins and proteoglycans. ECM proteins deposition and crosslink provides necessary biochemical and biophysical supports for cancer cell proliferation, migration and invasion 1, 2. Aberrant expression of ECM-related molecules and alteration of the balance of ECM signal will no doubt cause effect on cancer cell functions 3, 4. Thus, identifying molecules correlated with ECM regulation would help to develop novel diagnostic and therapeutic strategies for cancer treatment.

Among the various ECM components, collagens are the most abundant proteins, which were reported to modulate cancer cell migration, invasion 5, proliferation 6 and patient survival 7. The collagen family contains 28 members and can be divided into two groups: fibrillar collagen (type I, II, III, V, XI) and non-fibrillar collagen (type IV, VIII, X, IX, XII, XIV, XV, XVIII, XIX, XXI) 8. After synthesis in the rough endoplasmic reticulum, the collagen precursor went through a process of modifications including lysine hydroxylation for maturation. Hydroxylysine residue usually occurs in the Y position of the repeating Gly-X-Y (X and Y represent proline or hydroxylproline) motif 9, and is critical for the formation of collagen crosslink and glycosylation, stabilizing the newly formed collagen fibers and enhancing the stiffness of the matrix 10. Abnormal lysyl hydroxylation contributes to the progression of many collagen-related diseases, such as fibrosis and cancer 11. Therefore, modification events of maturation and the corresponding enzymes are important for proper function of collagen and related cancer cell biology.

Procollagen-lysine, 2-oxoglutarate 5-dioxygenase (PLOD) is the lysyl hydroxylase responsible for the lysyl hydroxylation of collagen 12. In human genome, three PLODs, namely PLOD1, 2 and 3, are identified and share high homology with an overall identity being 47% in protein sequences 9. PLOD1 and PLOD3 hydroxylate lysyl residues in the collagen triple helix, whereas PLOD2 hydroxylates lysyl residues in the telopeptides of collagen. In addition, PLOD3 has glycosylation activity towards monosaccharide or disaccharide attaching to collagen hydroxylysines 13. Mounting evidences are suggesting that increased collagen deposition and cross-linking can promote cancer progression by enhancing cancer cell proliferation, invasion and migration 14-17, and overexpression of each PLOD gene has been reported in different human cancers. Higher expression level of PLOD1 was observed in gastrointestinal carcinoma, and was related to carcinogenesis and clinical outcome 18. PLOD1 and PLOD2 were reportedly up-regulated in esophageal squamous-cell carcinoma and were associated with cell apoptosis, cell cycle and metastasis regulation 19, 20. Additionally, PLOD2 has been shown to promote metastasis in lung cancer, renal cell carcinoma and glioma 21-23. Overexpression of PLOD3 was observed in lung cancer and glioma, and was also associated with tumor progression and metastasis 24, 25. RNA interfering mediated down regulation of PLOD2 expression inhibited proliferation and invasion of bladder cancer cells 26. Therefore, the PLOD family genes could not only act as prognostic signatures but also become druggable targets for cancer therapy.

Gastric cancer (GC) is the fifth leading malignancy and the third leading cause of cancer-related deaths globally 27. The prognosis of GC is generally poor due to the fact that many patients had metastasis by the time of diagnosis and nearly inevitable tumor progression in spite of good initial treatment response, resulting in a five-year survival rate less than 10% 28. Recently, increased collagen deposition was found in GC compared to non-neoplastic tissues, and collagen width was demonstrated to be a powerful parameter for predicting 5-year overall survival of GC 29. Further studies of collagen related oncogenes would contribute to identifying novel molecular markers of GC progression and help develop new diagnostic and therapeutic strategies. With respect to PLOD family genes, the expression pattern and its relation with patient prognosis of individual PLODs in GC have recently been reported. High PLOD1 mRNA expression level was observed in gastric cancer and was associated with poor patient prognosis 18. PLOD2 was reported to promote cell invasiveness and migration in GC under hypoxia condition and lead to peritoneal dissemination and poor prognosis 30. PLOD3 mRNA was also up-regulated in GC tissues and cell lines, which was significantly correlated with larger tumor size and poor patient prognosis 31. In the current study, we investigated on the PLOD family genes based on different online databases. Study of individual PLOD family genes would be included, and we also highlight the prognostic values of co-expression of three PLOD genes for GC patients, and prediction of PLODs-related signaling pathways in GC.

## Methods

### ONCOMINE analysis

The mRNA expressions of PLOD1, 2 and 3 in different cancer types were analyzed using ONCOMINE gene expression array database 32 (www.oncomine.org), which is publicly accessible online. In this study, Student's *t* test was used to generate the *P*-value for expression differences of PLOD family genes between cancer specimens and normal controls. The fold change was defined as > 1 and the *P*-value was set up at 0.05.

### UALCAN analysis

UALCAN 33 is an interactive web-portal to perform in-depth analyses of level 3 RNA-seq and clinical data from 31 cancer type of The Cancer Genome Atlas (TCGA) database and it is publicly available at http://ualcan.path.uab.edu. We use it to analyze the relative expression of PLOD family genes between GC and normal samples and to compare the expression differences among various sub-groups based on cancer stage. The expression level was normalized as transcripts per million reads (TPM) and *P*-value < 0.01 calculated through Student's *t* test was considered as statistically significant.

### The Kaplan-Meier Plotter survival analysis

The prognostic value of expressions of PLOD family genes was analyzed using Kaplan-Meier Plotter online database, which is capable to assess the effect of multiple genes on survival using 18674 samples of diverse human malignancy, including 1065 GC patients 34. The examination probe ID used was as follows: 200827_at for PLOD1, 202620_s_at for PLOD2 and 202185_at for PLOD3. Each probe ID was entered into database with or without specific restrictions like cancer subtypes. The samples were divided into high and low expression groups by median value of mRNA expression of PLOD family genes. The correlations between gene expression and overall survival (OS), first progression (FP) and post progression survival (PPS) were validated by K-M survival curves and Log-rank test. The number-at-risk cases, hazard ratios (HRs), 95% confident intervals (CIs), and *P*-values were displayed accordingly. *P*-value < 0.05 was considered statistically significant.

### Functional protein interaction network construction

The Search Tool for the Retrieval of Interacting Genes (STRING) database 35 aims to construct functional protein association networks by consolidating known and predicted protein-protein association data for a large number of organisms. The STRING resource is available at http://string-db.org/. We use STRING database to establish a functional protein-protein interaction network among PLOD family genes. We selected the interactions pertaining to *Homo sapiens*, and 50 interactors were showed with a confidence score > 0.9.

### Gene function annotation and pathway enrichment analysis

Gene ontology (GO) analysis of the 50 interactors from STRING analysis was done by Database for Annotation, Visualization and Integrated Discovery (DAVID) online tool 36 (https://david.ncifcrf.gov/) for annotating genes and gene products and identifying characteristic biological attributes for high-throughput genome or transcriptome data, which includes 3 GO categories: cellular components, molecular functions, and biological processes. A GO category was considered significant enrichment when the *P*-value was < 0.05. Kyoto Encyclopedia of Genes and Genomes (KEGG) pathway enrichment analysis which evaluates the modules at the functional level was also executed by using DAVID for the selected interactors. *P*-value < 0.05 was set as the cut-off criterion and regarded as statistically significant.

### LinkedOmics analysis

The LinkedOmics database 37 (http://www.linkedomics.org/ login.php) is a Web-based platform for analyzing 32 TCGA cancer-associated multi-dimensional datasets. The *LinkFinder* module of LinkedOmics was used to study genes differentially expressed in correlation with PLOD1, 2 and 3 in the TCGA stomach adenocarcinoma (STAD) cohort (n=415). Results were analyzed statistically using Pearson's correlation coefficient. The *LinkFinder* also created statistical plots for individual genes. All results were graphically presented in volcano plots, or scatter plots.

## Results

### Expression levels of PLOD family genes are up-regulated in GC

**Figure 1:**
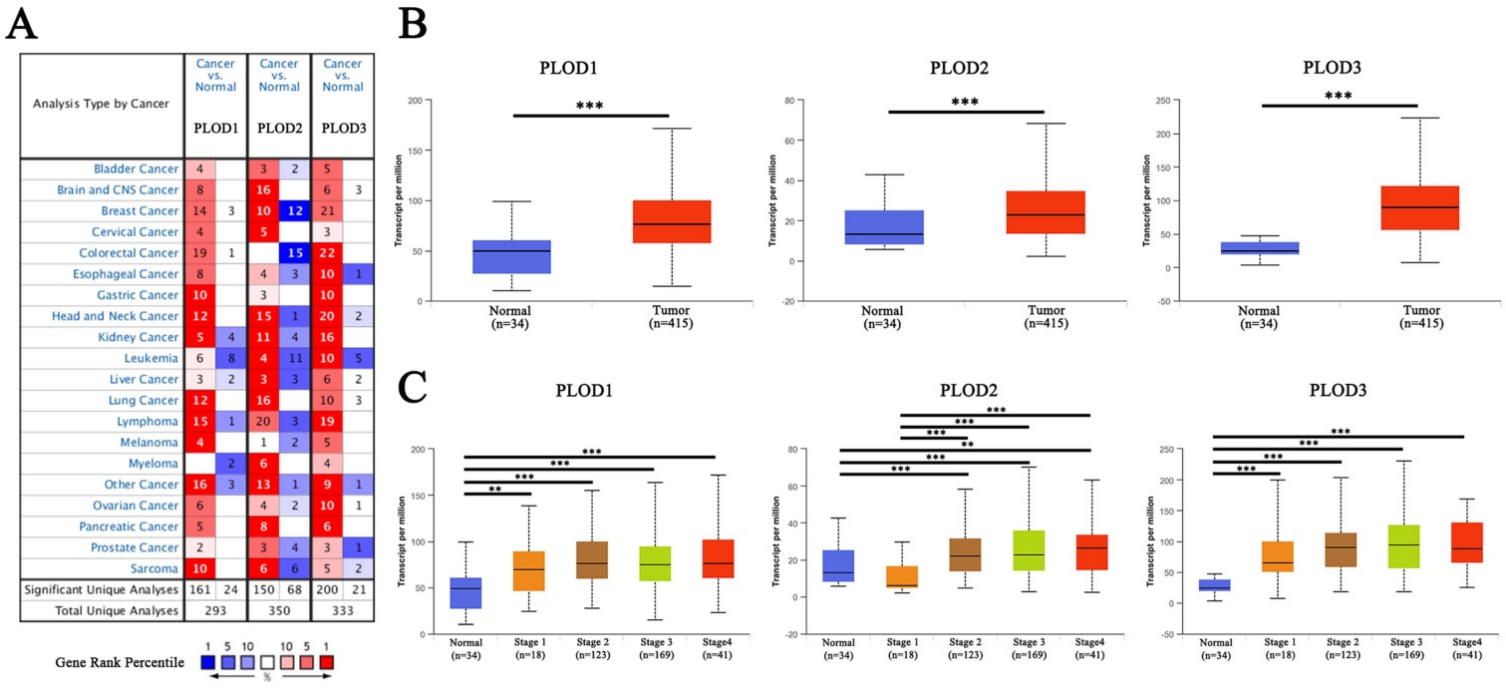
** Expression levels of PLOD family genes are up-regulated in GC.** (A) Expressions of PLOD1, 2 and 3 (cancer compared with normal tissue) were analyzed with ONCOMINE database. The graphic demonstrated the numbers of datasets with statistically significant mRNA overexpression (red) or downexpression (blue) of the target genes. The number in each cell represents the number of analyses that meet the threshold within those analysis and cancer types. The gene rank was analyzed by percentile of target gene in the top of all genes measured in each research. Cell color is determined by the best gene rank percentile for the analyses within the cell. (B) Boxplot showed the relative expression of PLOD1, 2 and 3 in GC tissues compared with non-tumor tissues from the STAD cohort of TCGA database. (C) Boxplot showed relative expression of PLOD1, 2 and 3 in normal individuals or in GC patients with different clinical stages. **P<0.01; ***P<0.001.

Firstly, we analyzed the expression levels of three PLOD genes in different kinds of human cancer using ONCOMINE database. All three PLOD genes showed a relatively up-regulated expression pattern in most of the cancer types. Noticeably, in gastric cancer, the expression levels of PLOD1, 2 and 3 were found to be up-regulated in 10, 3 and 10 analyses, respectively, with no down-regulated analyses under the threshold (Figure 1A). When comparing different GC subtype samples with normal tissue samples, PLOD1 mRNA expression showed significant elevation in gastric intestinal type adenocarcinoma in DErrico dataset, gastric cancer in Wang dataset, gastric adenocarcinoma in Cho dataset and gastric mixed adenocarcinoma in Chen dataset. PLOD2 only showed increased expression in gastric cancer in Wang dataset. As for PLOD3, the data showed broadly up-regulated expressions in various subtypes of GC from the listed 4 different datasets (Table 1). We then used UALCAN online portal to further verify the mRNA levels of PLOD genes in TCGA database. The result showed that all three PLOD genes were significantly up-regulated in the GC tissues compared to normal tissues (Figure 1B). When sorting the patients by clinical stages, all three PLOD genes were still significantly up-regulated in all stage subgroups compared with normal samples, except for PLOD2 which was down-regulated in stage 1 patients (Figure 1C). Taken together, the above results suggested that PLOD family genes were commonly up-regulated in GC, implying a potential role in GC development and progression.

### Elevated expressions of PLOD family genes predict poor clinical outcomes in GC patients

**Figure 2:**
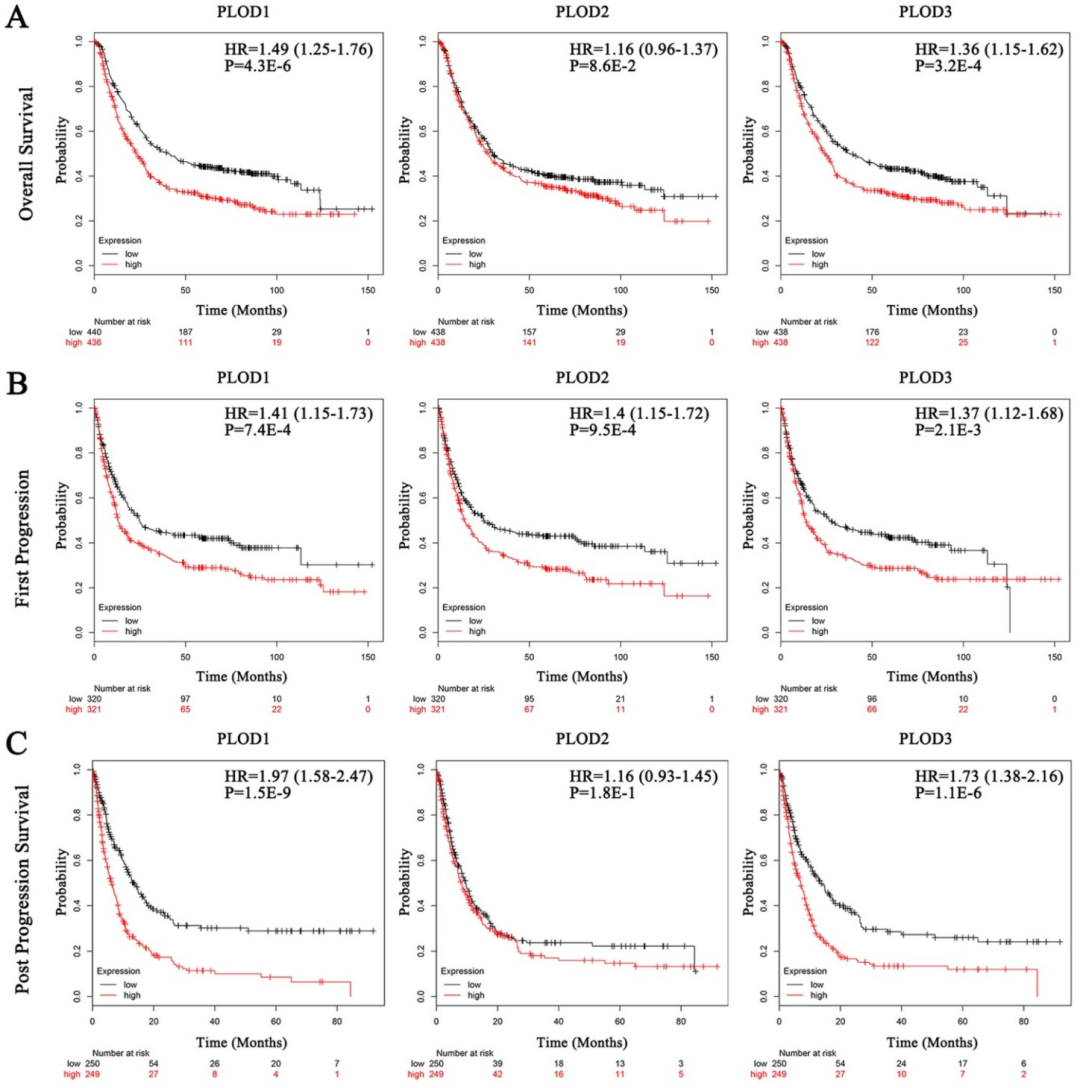
** Elevated expressions of PLOD family genes predict poor clinical outcomes in GC patients.** Each mRNA expression of PLOD1, 2 and 3 in tumor tissue was stratified into high or low expression using the median expression value as the cut-off point. Kaplan-Meier survival curves for (A) OS analysis with PLOD1 (left), PLOD2 (middle) and PLOD3 (right); (B) FP analysis with PLOD1 (left), PLOD2 (middle) and PLOD3 (right); (C) PPS analysis with PLOD1 (left), PLOD2 (middle) and PLOD3 (right), and the corresponding *P*-value for Log-rank test in the GC patients were showed.

**Figure 3:**
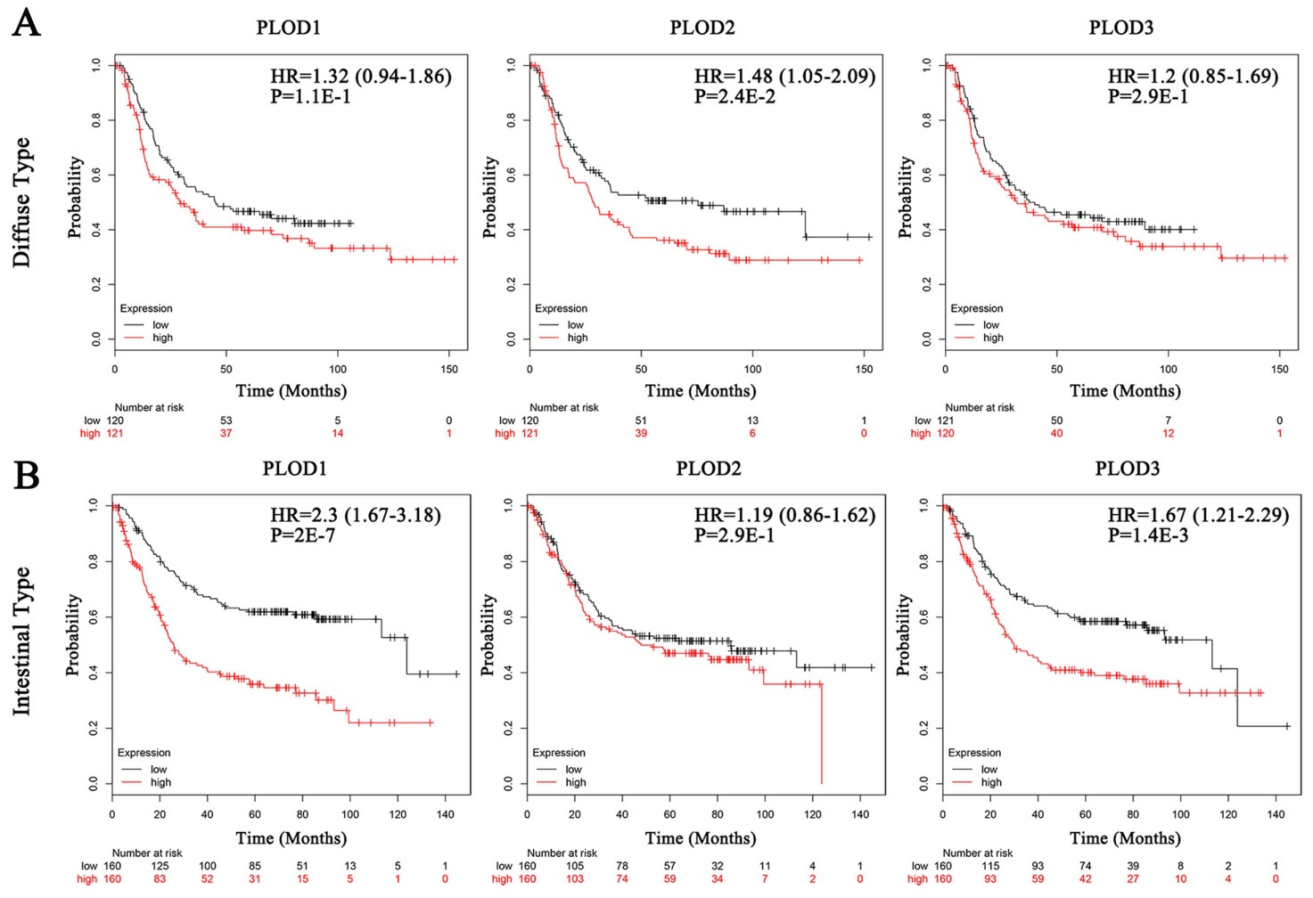
** The associations between mRNA expression of each PLOD gene in tumor tissue and OS of diffuse type or intestinal type GC patients.** Each mRNA expression of PLOD1, 2 and 3 in tumor tissue was stratified into high or low expression using the median expression value as the cut-off point. Kaplan-Meier survival curves (A) in diffuse type GC patients for OS analysis with PLOD1 (left), PLOD2 (middle) and PLOD3 (right) or (B) in intestinal type GC patients for OS analysis with PLOD1 (left), PLOD2 (middle) and PLOD3 (right), and the corresponding *P*-value for Log-rank test were showed.

Secondly, we analyzed the prognostic effects of PLOD gene expressions on GC patients with Kaplan-Meier Plotter database. We selected the median value to divide the samples into high and low expression groups, which can avoid high false discovery rate (FDR). The result showed that higher mRNA expressions of PLOD1 and PLOD3 were significantly associated with shorter OS, FP and PPS in GC. Otherwise, higher mRNA expression of PLOD2 could only predict shorter FP but not OS and PPS (Figure 2A-C). Interestingly, when sorting the samples by different GC subtypes, we found that high mRNA expressions of PLOD1 and PLOD3 were significantly correlated with shorter OS in intestinal type but not in diffuse type GC. Conversely, high mRNA expression of PLOD2 was only significantly correlated with shorter OS in diffuse type but not in intestinal type GC (Figure 3A-B). Further analysis had also confirmed that only high PLOD2 expression, but not PLOD1 or PLOD3, was significantly correlated with both shorter FP and PPS in diffuse type GC (Supplementary Figure 1A-B and 2A-B). Taken together, the above results suggested that PLOD family genes have prognostic values in GC patients and PLOD2 may exert this predictive function especially in diffuse type GC.

### Co-overexpression of PLOD family genes is a more powerful prognostic parameter for GC patient survival

**Figure 4:**
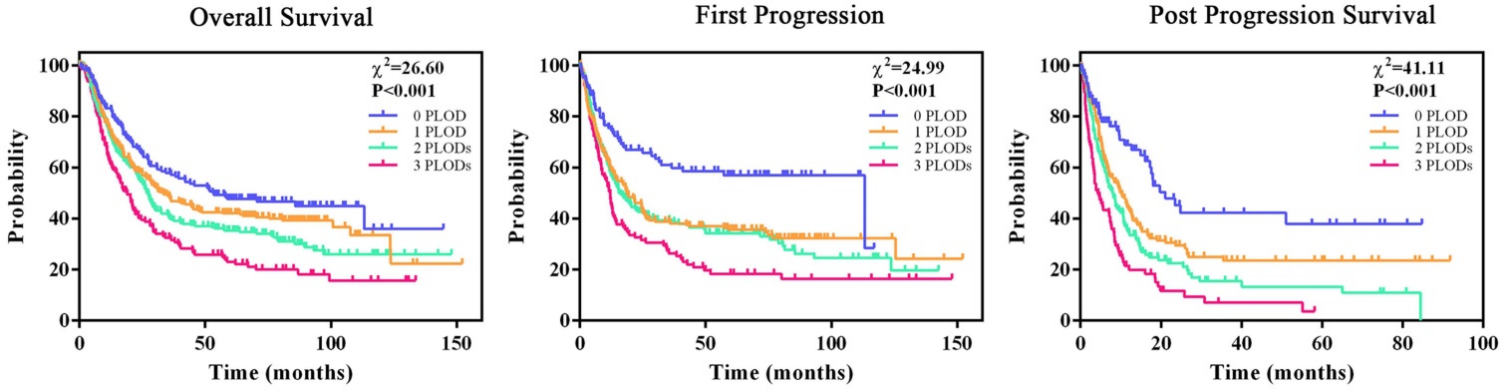
** Co-overexpression of PLOD family genes is a more powerful prognostic parameter for GC patient survival.** All GC patients were stratified into four groups according to the number of overexpressing PLOD genes. Kaplan-Meier curves by the four groups for OS analysis (left, 0 overexpressing PLOD, n=150; 1 overexpressing PLOD, n=287; 2 overexpressing PLODs, n=291; 3 overexpressing PLODs, n=146), FP analysis (middle, 0 overexpressing PLOD, n=106; 1 overexpressing PLOD, n=221; 2 overexpressing PLODs, n=198; 3 overexpressing PLODs, n=114) and PPS analysis (right, 0 overexpressing PLOD, n=86; 1 overexpressing PLOD, n=165; 2 overexpressing PLODs, n=162; 3 overexpressing PLODs, n=86), and the corresponding *P*-value for Log-rank test were showed.

As increased expression of any PLOD gene had a trend to be associated with poor survival rate of GC patients, we hypothesized that co-overexpression of PLOD family genes could a better prognostic parameter for GC. Kaplan-Meier analysis showed that GC patients from TCGA database whose tumors co-overexpressed more PLOD genes had a significant shorter median OS, FP and PPS time compared with those whose tumors co-overexpressed fewer PLOD genes (Figure 4). The median survival time of 0 PLOD, 1 PLOD, 2 PLODs and 3 PLODs groups was 53.4, 33.3, 27.0 and 19.0 months for OS analysis, 113.2, 18.6, 16.1 and 12.0 months for FP analysis, 21.0, 10.2, 7.9 and 4.3 months for PPS analysis, respectively. Consistently, we found an increasing HR values for OS, FP and PPS analysis when more PLOD genes were co-expressing in GC patients (Table 2). Taken together, the above results suggested that the combinatorial use of expressions of more PLOD genes may be a reliable prognostic indicator for GC patients.

### Functional protein interaction network of PLOD family genes

**Figure 5:**
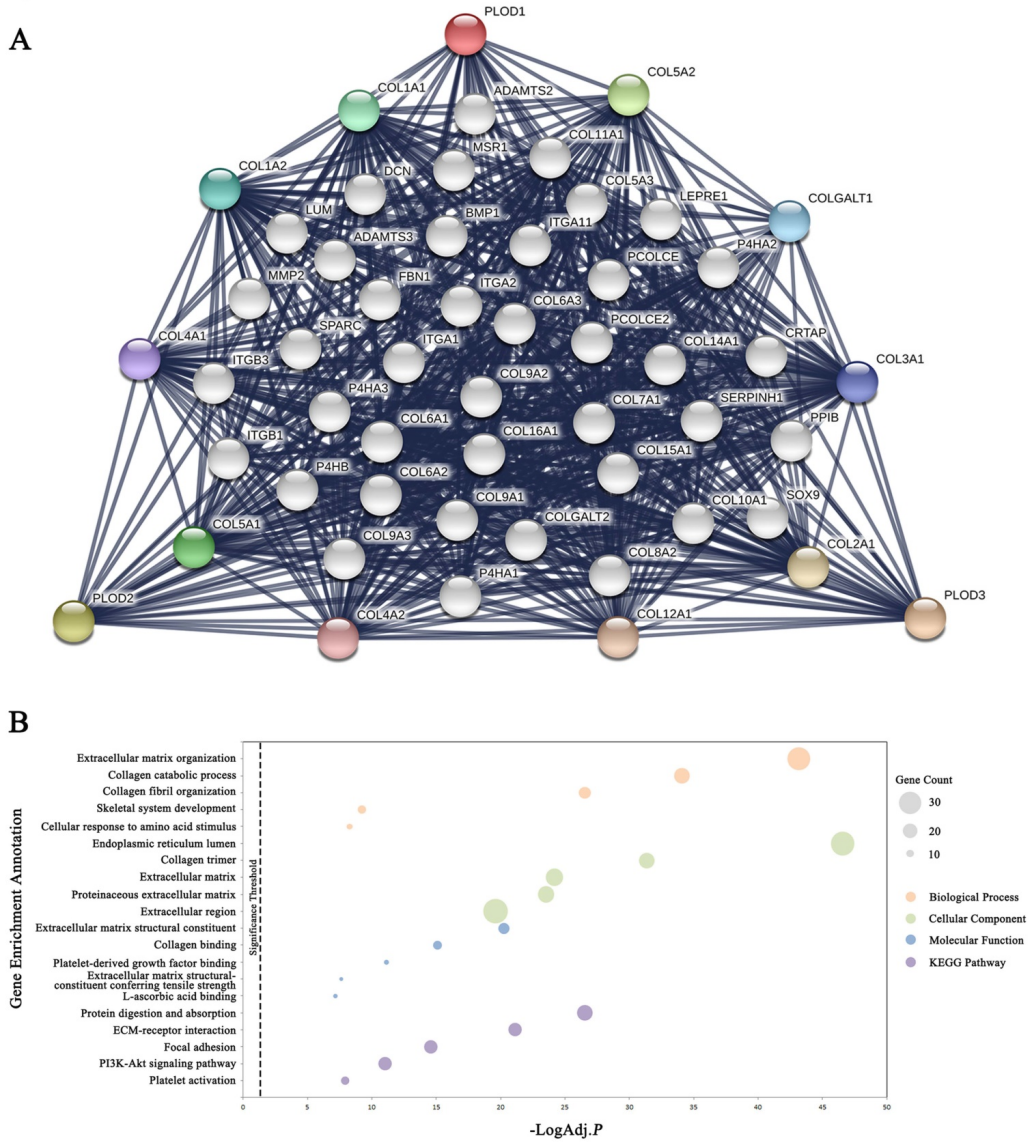
** Functional protein interaction network of PLOD family genes.** (A) Protein interaction network of 50 functional partners with confidence score > 0.9 based on STRING database. PLOD1, 2 and 3 are the seed genes. Ten interacting partners with the highest confident scores were colored and placed in the outer shell. The other forty interacting partners were grey and placed in the inner shell. The blue lines represent the correlation between proteins and the thickness of the lines indicates the strength of data support. (B) The bubble diagram displayed the enrichment results of the 50 functional interacting partners. The top 5 enriched categories for Biological Process, Cellular Component, Molecular Function and KEGG Pathway analysis were showed.

To explore the possible PLODs-mediated signaling pathways in GC, we began with the STRING database to find out the interacting protein network of PLOD family. The outer shell of the network included 10 functional partners with highest interacting confidence score, namely COL5A2, COL5A1, COL1A1, COL1A2, COLGALT1, COL3A1, COL4A1, COL4A2, COL12A1 and COL2A1. The inner shell included other 40 functional partners all with an interacting confidence score above 0.9 (Figure 5A). Further analysis of enriched GO terms with DAVID using these 50 functional interacting partners indicated that these proteins localized mainly to the endoplasmic reticulum lumen, collagen trimer and extracellular matrix. Biological process analysis showed that these proteins are primarily involved in extracellular matrix organization, collagen catabolic process and collagen fibril organization. Molecular function included extracellular matrix structural constituent, collagen binding and platelet-derived growth factor binding. KEGG pathway analysis showed enrichment in the protein digestion and absorption, ECM-receptor interaction and focal adhesion (Figure 5B and Supplementary Table 1). Taken together, the above results indicated that PLOD family genes were mainly involved in regulating the collagen metabolism and extracellular matrix constitution.

### Signaling modules correlated with PLOD family genes in GC

**Figure 6:**
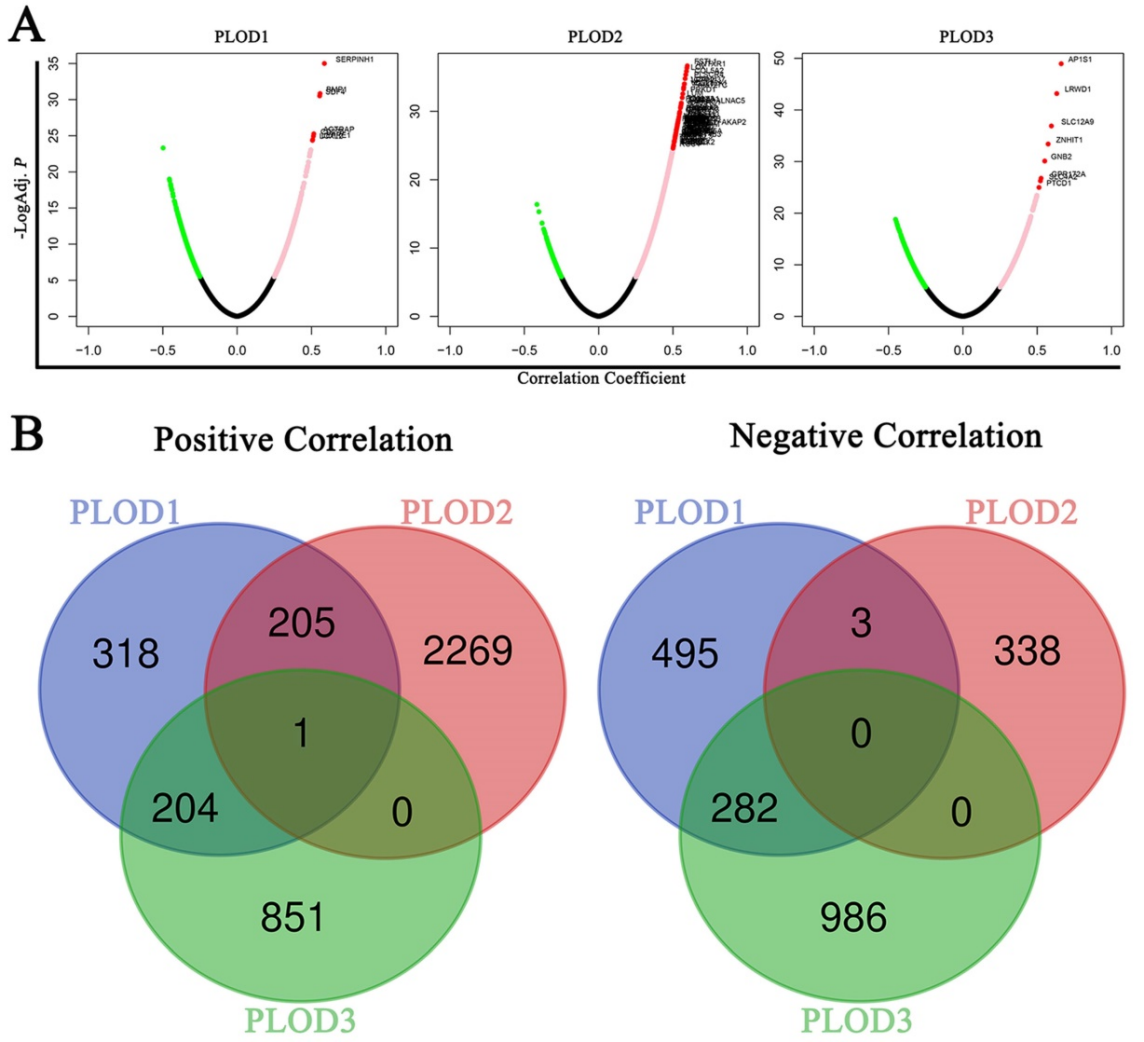
** Signaling modules correlated with PLOD family genes in GC.** (A) Volcano plot showed the co-expression genes correlated with PLOD1, 2 and 3 in the STAD cohort from TCGA database. A Pearson correlation test was used to analyze between PLOD genes and genes differentially expressed in GC. Green dots, pink dots and red dots showed genes with a correlation coefficient < -0.25, > 0.25 and < 0.5, and > 0.5, respectively. (B) Left, Venn diagram showed the positively (left) and negatively (right) co-expressing genes correlated with PLOD1, 2 and 3.

To further verify the PLODs-related molecules functioning in GC, We used the *Function* module of LinkedOmics database to analyze mRNA sequencing data from 415 GC patients in the TCGA. As shown in the volcano plot, there are 728, 2475 and 1056 significantly positive correlated genes (pink dots and red dots) with PLOD1, PLOD2 and PLOD3 in GC samples, respectively. Whereas, 780, 341 and 1268 genes showed significant negative correlations (green dots) with PLOD1, PLOD2 and PLOD3 in GC samples, respectively (Figure 6A and Supplementary Table 2). To find out the concurrent regulatory molecules, Venn diagram was used to show that only SHC1 (Src homology and collagen homology 1) gene was positively co-expressed with PLOD1, 2 and 3, and no gene was negatively co-expressed with PLOD1, 2 and 3 in GC (Figure 6B and Supplementary Figure 3A-C). Taken together, the above results suggested that SHC1 may be a regulatory module mediating PLOD's effect on GC.

## Discussion

In the current study, we demonstrated that all PLOD family genes were highly expressed in GC. High expression levels of PLOD1 and PLOD3 were associated with shorter OS, FP and PPS while high expression level of PLOD2 was only associated with shorter FP in all GC patients. When sorting the GC patients by pathological subtypes, only high PLOD2 expression had significant correlation with shorter OS, FP and PPS in the diffuse type GC patients. We further proved that the combinatorial use of expressions of more PLOD genes could be a superior prognostic indicator for GC patients. Pathway analysis confirmed that PLOD family genes mainly participate in regulating the collagen metabolism and extracellular matrix constitution, and SHC1 could be the regulatory module mediating PLOD's effect on GC. Therefore, we propose that individual PLOD genes or PLOD family genes as a whole could be potential prognostic biomarkers for GC.

Several studies have focused on the prognostic role of individual PLOD gene in GC 18, 29, 30, which is consistent to our study that to some extent each PLOD gene indeed is of prognostic value. Here we identified a more powerful prognostic indicator that could be of clinical significance by combining the expression of all PLOD family genes for the prediction of OS as well as FP and PPS in GC patients. Moreover, after analysis of the pathological subtypes, PLOD2 seemed to be a more sensitive prognostic parameter to the diffuse type GC whose characteristics include thick stromal fibrosis and high levels of transforming growth factor β (TGF-β) 38. Different from PLOD1 and PLOD3, PLOD2 has a preferential role in the formation of stabilized collagen crosslinks 39, which contribute to a fibrosis-prone microenvironment. Additionally, it was also reported that activating TGF-β signaling induced PLOD2 transcription via histone modification of *PLOD2* promoter region 40. Therefore, PLOD2 was speculated to be important for the development and progression specifically in the diffuse type GC. However, the exact mechanism still warrants further investigation.

Pathway analysis revealed that PLOD family genes exert their functions mainly on the collagen metabolism and extracellular matrix constitution, which was supported by dozens of publications. However, our further study indicated that SHC1, a well-known cellular adaptor protein, could be the signaling transduction module mediating PLODs' effect on GC. SHC1 is the founding member of the Shc family of adaptor proteins, which share similar topographic domains, CH2 (collagen homology 2)-PTB (phosphotyrosine binding)-CH1 (collagen homology 1)-SH2 (Src homology 2), in their protein sequences 41. The deregulation of Shc proteins has been implicated in various diseases, including cancers, indicating their important regulation on cellular physiology 41, 42. The canonical role of Shc family proteins is to sense the signals at the cellular membrane, by transmitting them from the ECM to internal environment and eliciting specific responses, generally via mitogen-activated protein kinases (MAPK) and phosphoinositide-3-kinase/Akt signaling pathways 41, 43. Among those membranous receptors that locate upstream of Shc family proteins, integrins are the kind of receptors that have been reported to transmit ECM signals in couple with tyrosine kinases to regulate cellular proliferation, migration and angiogenesis. Sweet DT. *et al* showed that SHC1 was required for activation of the Akt pathway downstream of both integrin and VEGF signaling, by integrating the signals from these 2 receptors when cells are grown on fibronectin 44. Galvagni F. *et al* demonstrated that cell adhesion to extracellular matrix induced the integrin-mediated VEGFR-3 phosphorylation and recruitment of SHC1, subsequently promoting downstream signaling for cell proliferation and migration 45. Integrin-SHC1 signal axis had effect on collagen-I activating up-regulation of N-cadherin to promote tumor growth, invasion and metastasis in human pancreatic cancer 46, 47. SHC1 was also necessary to link integrins to Ras-ERK pathway, thus promoting cell cycle progression and anchorage-dependent cell growth via caveolin-1-Fyn mediated phosphorylation 48. The above studies again suggested that ECM may play important role on cellular functions through integrin-SHC1 signaling transduction. Since the enzymatic activity of PLOD1, 2 and 3 has great impact on the component and structure of the ECM, and SHC1 was positively co-expressed with PLODs in GC, we therefore hypothesized that PLODs synergized with SHC1 and participated in integrin-SHC1 signaling cascades to regulate GC cell proliferation and migration by modifying the ECM (Supplementary Figure 3D). However, further investigations are needed.

There are limitations in our study. Our study of prognostic prediction was mainly based on the STAD patient cohort from TCGA database. Although laboratory experiment procedures could remain consistent from one database, more independent GC patient cohorts are warranted to confirm our findings. To the best of our knowledge, this is the first report where we evaluated the prognostic values of all the PLOD genes in GC patients. Our study provided a whole image of the correlation between expressions of PLOD family genes and patient prognosis in one cancer type, which made the case more convincing that PLOD genes plays an important role in the development and progression of GC. Secondly, only the mRNA expression levels of PLOD genes were confirmed to be significantly correlated with prognosis of GC patients in this study. Indeed, we also detected the protein expressions of three PLODs on a tissue microarray composed of 40 pairs of GC tissues and matched adjacent normal tissues. Our primary results showed that protein expressions of PLOD1 and PLOD3 were decreased in GC tissues compared to normal tissues, and expression of PLOD2 protein showed no difference between cancer and normal tissues, which was somewhat contradictory to our findings that mRNA expressions of PLODs were elevated in GC patients in this study (data not shown). However, it has been reported that the correlation between mRNA and protein abundances in the cell was poor and there were presumably reasons for this discrepancy 49, 50. First, many complicated and varied post-transcriptional mechanisms involved in turning mRNA into protein affect mRNA-protein correlation, such as RNA secondary structure, codon bias, ribosomal density, protein half-lives or existence of untranslated RNA species; second, there is a significant amount of experiment error and noise in both mRNA and protein measurement that accounts for the poor mRNA-protein correlation. However, the exact mechanism of the discrepancy between mRNA and protein expressions of PLODs in GC and whether protein expressions of PLODs could be used as prognostic markers in GC patients need further investigation.

In conclusion, the current study has demonstrated that overexpressing PLOD genes are associated with poor prognosis of GC patients and combinatory use of all PLOD genes could be a superior prognostic indicator for GC patients. SHC1 could be the regulatory module mediating PLOD's effect on GC by integrating the ECM and intracellular signaling events.

## Supplementary Material

Supplementary figures and tables.

## Figures and Tables

**Table 1:** Significantly upregulated mRNA expressions of PLOD family genes in GC from ONCOMINE database^*^

PLOD Family Genes	Fold Change	*P* Value	Dataset	Group Comparison
PLOD1				
	2.030	3.74E-11	DErrico Gastric	Gastric Intestinal Type Adenocarcinoma *vs.* Normal
	1.775	4.00E-03	Wang Gastric	Gastric Cancer *vs.* Normal
	1.642	7.00E-03	Cho Gastric	Gastric Adenocarcinoma *vs.* Normal
	1.611	2.27E-04	Chen Gastric	Gastric Mixed Adenocarcinoma *vs.* Normal
PLOD2				
	1.605	1.30E-02	Wang Gastric	Gastric Cancer *vs.* Normal
PLOD3				
	2.672	1.25E-11	DErrico Gastric	Gastric Intestinal Type Adenocarcinoma *vs.* Normal
	1.799	1.60E-02	DErrico Gastric	Diffuse Gastric Adenocarcinoma *vs.* Normal
	2.139	3.49E-11	Cho Gastric	Diffuse Gastric Adenocarcinoma *vs.* Normal
	2.037	1.86E-07	Cho Gastric	Gastric Intestinal Type Adenocarcinoma *vs.* Normal
	1.995	5.00E-03	Cho Gastric	Gastric Adenocarcinoma *vs.* Normal
	1.834	2.26E-04	Cho Gastric	Gastric Mixed Adenocarcinoma *vs.* Normal
	2.071	6.58E-06	Chen Gastric	Gastric Mixed Adenocarcinoma *vs.* Normal
	1.881	3.08E-12	Chen Gastric	Gastric Intestinal Type Adenocarcinoma *vs.* Normal
	1.541	4.96E-10	Cui Gastric	Gastric Cancer *vs.* Normal

*Only datasets that meet the criteria *P* value < 0.05 and fold change > 1.5 are listed.

**Table 2:** Correlation between the number of PLOD genes overexpressed and the prognosis in GC patients

Factor	HR	95% CI	*P*-Value
**OS Prediction on PLODs Co-overexpression**			
0 PLOD	Reference		
1 PLOD	1.240	0.957-1.607	0.079
2 PLODs	1.473	1.149-1.888	0.003*
3 PLODs	2.135	1.590-2.869	< 0.001*
**FP Prediction on PLODs Co-overexpression**			
0 PLOD	Reference		
1 PLOD	1.716	1.259-2.338	< 0.001*
2 PLODs	1.807	1.320-2.476	< 0.001*
3 PLODs	2.668	1.874-3.783	< 0.001*
**PPS Prediction on PLODs Co-overexpression**			
0 PLOD	Reference		
1 PLOD	1.641	1.154-2.335	0.006*
2 PLODs	2.088	1.499-2.908	< 0.001*
3 PLODs	3.351	2.257-4.975	< 0.001*

*P< 0.05.

## Abbreviations

CH1: collagen homology 1
CH2: collagen homology 2
CI: confident interval
DAVID: Database for Annotation, Visualization and Integrated Discovery
ECM: extracellular matrix
FDR: false discovery rate
FP: first progression
GC: gastric cancer
GO: gene ontology
HR: hazard ratio
KEGG: Kyoto Encyclopedia of Genes and Genomes
MAPK: mitogen-activated protein kinases
OS: overall survival
PLOD: procollagen-lysine, 2-oxoglutarate 5-dioxygenase
PPS: post progression survival
PTB: phosphotyrosine binding
SH2: Src homology 2
SHC1: Src homology and collagen homology 1
STAD: stomach adenocarcinoma
STRING: Search Tool for the Retrieval of Interacting Genes
TCGA: The Cancer Genome Atlas
TGF-β: transforming growth factor β
TPM: transcripts per million reads
