# Supplementary material for: Overexpressing PLOD family genes predict poor prognosis in gastric cancer
Source: J Cancer. 2020 Jan 1;11(1):121–31. doi: 10.7150/jca.35763 (PMC6930397; doi:10.7150/jca.35763)

Supplementary Table 1. Summary of significantly enriched GO annotations of PLODs related network

| Description | Gene Count | Adjusted *P*-Value | Enriched Genes |
|---|---|---|---|
| ***Biological Process*** | | | |
| Extracellular matrix organization | 31 | 6.70E-44 | SOX9; COL1A1; COL1A2; COL2A1; COL3A1; COL4A1; COL4A2; COL9A1; COL9A2; COL9A3; COL5A1; COL5A2; COL5A3; COL6A1; COL6A2; COL6A3; COL7A1; COL8A2; COL10A1; COL11A1; COL14A1; COL16A1; DCN; FBN1; ITGA1; ITGA11; ITGA2; ITGB1; ITGB3; LUM; SPARC |
| Collagen catabolic process | 21 | 8.40E-35 | ADAMTS2; ADAMTS3; COL1A1; COL1A2; COL2A1; COL3A1; COL4A1; COL4A2; COL5A1; COL5A2; COL5A3; COL6A1; COL6A2; COL6A3; COL7A1; COL8A2; COL10A1; COL11A1; COL12A1; COL15A1; MMP2 |
| Collagen fibril organization | 16 | 2.90E-27 | ADAMTS2; ADAMTS3; COL1A1; COL1A2; COL2A1; COL3A1; COL5A1; COL5A2; COL5A3; COL11A1; COL12A1; COL14A1; LUM; PLOD3; P4HA1; SERPINH1 |
| Skeletal system development | 11 | 6.20E-10 | SOX9; BMP1; COL1A1; COL1A2; COL2A1; COL3A1; COL9A2; COL5A2; COL10A1; COL12A1; FBN1 |

| | | | |
|---|---|---|---|
| Cellular response to amino acid stimulus | 8 | 5.60E-09 | COL1A1; COL1A2; COL3A1; COL4A1; COL5A2; COL6A1; COL16A1; MMP2 |
| ***Cellular Component*** | | | |
| Endoplasmic reticulum lumen | 32 | 2.70E-47 | CRTAP; COLGALT1; COLGALT2; COL1A1; COL1A2; COL2A1; COL3A1; COL4A1; COL4A2; COL9A1; COL9A2; COL9A3; COL5A1; COL5A2; COL5A3; COL6A1; COL6A2; COL6A3; COL7A1; COL8A2; COL10A1; COL11A1; COL12A1; COL14A1; COL15A1; COL16A1; PPIB; P4HA1; P4HA2; P4HA3; P4HB; SERPINH1 |
| Collagen trimer | 21 | 4.50E-32 | COL1A1; COL1A2; COL2A1; COL3A1; COL9A2; COL5A1; COL5A2; COL6A1; COL6A2; COL6A3; COL7A1; COL8A2; COL10A1; COL11A1; COL12A1; COL14A1; COL15A1; MSR1; PCOLCE; P4HA1; SERPINH1 |
| Extracellular matrix | 23 | 6.80E-25 | ADAMTS3; COL1A1; COL1A2; COL2A1; COL3A1; COL4A1; COL4A2; COL5A1; COL5A2; COL6A1; COL6A2; COL6A3; COL7A1; COL8A2; COL12A1; COL14A1; COL15A1; DCN; FBN1; LUM; MMP2; PCOLCE; P4HB |
| Proteinaceous extracellular matrix | 22 | 2.90E-24 | ADAMTS2; ADAMTS3; BMP1; CRTAP; COL1A2; COL9A1; |

| | | | |
|---|---|---|---|
| | | | COL9A2; COL9A3; COL5A1; COL5A2; COL6A2; COL6A3; COL8A2; COL10A1; COL11A1; COL14A1; COL15A1; COL16A1; FBN1; LUM; MMP2; SPARC |
| Extracellular region | 33 | 2.60E-20 | ADAMTS2; ADAMTS3; BMP1; COL1A1; COL1A2; COL2A1; COL3A1; COL4A1; COL4A2; COL9A1; COL9A2; COL9A3; COL5A1; COL5A2; COL5A3; COL6A1; COL6A2; COL6A3; COL7A1; COL8A2; COL10A1; COL11A1; COL12A1; COL14A1; COL15A1; COL16A1; DCN; FBN1; LUM; MMP2; PCOLCE2; P4HB; SPARC |
| ***Molecular Function*** | | | |
| Extracellular matrix structural constituent | 15 | 5.60E-21 | COL1A1; COL1A2; COL2A1; COL3A1; COL4A1; COL4A2; COL5A1; COL5A2; COL5A3; COL8A2; COL11A1; COL14A1; COL15A1; FBN1; LUM |
| Collagen binding | 12 | 7.90E-16 | COL5A3; COL14A1; DCN; ITGA1; ITGA11; ITGA2; LUM; PPIB; PCOLCE2; PCOLCE; SPARC; SERPINH1 |
| Platelet-derived growth factor binding | 7 | 7.30E-12 | COL1A1; COL1A2; COL2A1; COL3A1; COL4A1; COL5A1; COL6A1 |
| Extracellular matrix structural constituent conferring tensile strength | 5 | 2.40E-08 | COL2A1; COL9A1; COL9A2; COL9A3; COL12A1 |

| | | | |
|---|---|---|---|
| L-ascorbic acid binding | 6 | 6.90E-08 | PLOD1; PLOD2; PLOD3; P4HA1; P4HA2; P4HA3 |
| ***KEGG Pathway*** | | | |
| Protein digestion and absorption | 21 | 2.90E-27 | COL1A1; COL1A2; COL2A1; COL3A1; COL4A1; COL4A2; COL9A1; COL9A2; COL9A3; COL5A1; COL5A2; COL5A3; COL6A1; COL6A2; COL6A3; COL7A1; COL10A1; COL11A1; COL12A1; COL14A1; COL15A1 |
| ECM-receptor interaction | 18 | 7.80E-22 | COL1A1; COL1A2; COL2A1; COL3A1; COL4A1; COL4A2; COL5A1; COL5A2; COL5A3; COL6A1; COL6A2; COL6A3; COL11A1; ITGA1; ITGA11; ITGA2; ITGB1; ITGB3 |
| Focal adhesion | 18 | 2.80E-15 | COL1A1; COL1A2; COL2A1; COL3A1; COL4A1; COL4A2; COL5A1; COL5A2; COL5A3; COL6A1; COL6A2; COL6A3; COL11A1; ITGA1; ITGA11; ITGA2; ITGB1; ITGB3 |
| PI3K-Akt signaling pathway | 18 | 9.40E-12 | COL1A1; COL1A2; COL2A1; COL3A1; COL4A1; COL4A2; COL5A1; COL5A2; COL5A3; COL6A1; COL6A2; COL6A3; COL11A1; ITGA1; ITGA11; ITGA2; ITGB1; ITGB3 |
| Platelet activation | 11 | 1.20E-08 | COL1A1; COL1A2; COL2A1; COL3A1; COL5A1; COL5A2; COL5A3; COL11A1; ITGA2; ITGB1; ITGB3 |

Supplementary Table 2. Summary of genes correlated to PLODs in GC from TCGA database*

| PLOD Protein Genes | Correlated Gene | Correlation Coefficient | *P*-Value | Adj. *P*-Value |
|---|---|---|---|---|
| PLOD1 | SERPINH1 | 5.87E-01 | 1.01E-39 | 1.02E-35 |
| PLOD1 | LEPRE1 | 5.09E-01 | 1.14E-28 | 3.28E-25 |
| PLOD1 | LOXL2 | 5.07E-01 | 1.71E-28 | 4.32E-25 |
| PLOD1 | BMP1 | 5.58E-01 | 2.22E-35 | 1.50E-31 |
| PLOD1 | CD276 | 5.13E-01 | 3.39E-29 | 1.14E-25 |
| PLOD1 | SDF4 | 5.55E-01 | 6.13E-35 | 3.10E-31 |
| PLOD1 | AGTRAP | 5.16E-01 | 1.32E-29 | 5.34E-26 |
| PLOD2 | LEPRE1 | 5.18E-01 | 7.67E-30 | 2.43E-27 |
| PLOD2 | CERCAM | 5.25E-01 | 8.48E-31 | 3.18E-28 |
| PLOD2 | COL5A1 | 5.24E-01 | 1.16E-30 | 4.20E-28 |
| PLOD2 | COL5A2 | 5.86E-01 | 1.12E-39 | 4.52E-36 |
| PLOD2 | INHBA | 5.36E-01 | 2.87E-32 | 1.82E-29 |
| PLOD2 | P4HA3 | 5.24E-01 | 1.15E-30 | 4.20E-28 |
| PLOD2 | COL1A2 | 5.46E-01 | 1.40E-33 | 1.24E-30 |
| PLOD2 | SPARC | 5.29E-01 | 2.36E-31 | 9.94E-29 |
| PLOD2 | COL12A1 | 5.73E-01 | 1.43E-37 | 2.90E-34 |
| PLOD2 | CHSY3 | 5.56E-01 | 5.16E-35 | 6.29E-32 |
| PLOD2 | MFAP2 | 5.23E-01 | 1.45E-30 | 5.06E-28 |
| PLOD2 | CTHRC1 | 5.52E-01 | 2.05E-34 | 1.98E-31 |
| PLOD2 | NID2 | 5.08E-01 | 1.43E-28 | 3.61E-26 |
| PLOD2 | LOX | 5.90E-01 | 2.82E-40 | 1.43E-36 |
| PLOD2 | COL3A1 | 5.56E-01 | 5.29E-35 | 6.29E-32 |
| PLOD2 | MMP2 | 5.13E-01 | 3.33E-29 | 9.10E-27 |
| PLOD2 | FN1 | 5.56E-01 | 4.47E-35 | 6.03E-32 |
| PLOD2 | VCAN | 5.77E-01 | 3.70E-38 | 9.35E-35 |
| PLOD2 | NTM | 5.36E-01 | 3.16E-32 | 1.88E-29 |

| | | | | |
|---|---|---|---|---|
| PLOD2 | COL6A3 | 5.36E-01 | 2.98E-32 | 1.83E-29 |
| PLOD2 | TMEM45A | 5.17E-01 | 1.07E-29 | 3.24E-27 |
| PLOD2 | FNDC1 | 5.30E-01 | 2.28E-31 | 9.80E-29 |
| PLOD2 | FAP | 5.32E-01 | 1.08E-31 | 5.09E-29 |
| PLOD2 | EFEMP2 | 5.17E-01 | 8.80E-30 | 2.74E-27 |
| PLOD2 | NOX4 | 5.24E-01 | 1.22E-30 | 4.34E-28 |
| PLOD2 | PDPN | 5.34E-01 | 6.06E-32 | 3.40E-29 |
| PLOD2 | SULF1 | 5.31E-01 | 1.51E-31 | 6.78E-29 |
| PLOD2 | MEIS3 | 5.04E-01 | 4.08E-28 | 1.01E-25 |
| PLOD2 | ANTXR1 | 5.94E-01 | 6.91E-41 | 4.66E-37 |
| PLOD2 | TIMP2 | 5.55E-01 | 7.91E-35 | 8.89E-32 |
| PLOD2 | GFPT2 | 5.44E-01 | 2.67E-33 | 2.07E-30 |
| PLOD2 | SRPX2 | 5.09E-01 | 8.74E-29 | 2.26E-26 |
| PLOD2 | CDH11 | 5.29E-01 | 2.59E-31 | 1.07E-28 |
| PLOD2 | FSTL1 | 5.97E-01 | 2.15E-41 | 2.17E-37 |
| PLOD2 | FBN1 | 5.19E-01 | 5.22E-30 | 1.68E-27 |
| PLOD2 | C14orf37 | 5.77E-01 | 3.50E-38 | 9.35E-35 |
| PLOD2 | CDH2 | 5.16E-01 | 1.25E-29 | 3.60E-27 |
| PLOD2 | GPX8 | 5.43E-01 | 3.57E-33 | 2.49E-30 |
| PLOD2 | VGLL3 | 5.72E-01 | 1.77E-37 | 3.26E-34 |
| PLOD2 | C13orf33 | 5.38E-01 | 1.92E-32 | 1.25E-29 |
| PLOD2 | LUM | 5.62E-01 | 7.00E-36 | 1.01E-32 |
| PLOD2 | COPZ2 | 5.23E-01 | 1.68E-30 | 5.66E-28 |
| PLOD2 | THBS1 | 5.40E-01 | 9.57E-33 | 6.45E-30 |
| PLOD2 | RAB31 | 5.52E-01 | 2.03E-34 | 1.98E-31 |
| PLOD2 | SDC2 | 5.50E-01 | 3.57E-34 | 3.28E-31 |
| PLOD2 | PRKD1 | 5.66E-01 | 1.73E-36 | 2.70E-33 |
| PLOD2 | RAI14 | 5.45E-01 | 1.64E-33 | 1.37E-30 |
| PLOD2 | PDGFC | 5.27E-01 | 5.16E-31 | 2.01E-28 |

| | | | | |
|---|---|---|---|---|
| PLOD2 | LRP12 | 5.76E-01 | 4.76E-38 | 1.07E-34 |
| PLOD2 | LARP6 | 5.04E-01 | 3.57E-28 | 8.91E-26 |
| PLOD2 | ST6GALNAC5 | 5.54E-01 | 1.04E-34 | 1.11E-31 |
| PLOD2 | SCHIP1 | 5.23E-01 | 1.58E-30 | 5.40E-28 |
| PLOD2 | FAM127C | 5.71E-01 | 3.33E-37 | 5.61E-34 |
| PLOD2 | RGS4 | 5.01E-01 | 1.04E-27 | 2.44E-25 |
| PLOD2 | MYH10 | 5.23E-01 | 1.80E-30 | 5.97E-28 |
| PLOD2 | GLT8D2 | 5.35E-01 | 3.75E-32 | 2.17E-29 |
| PLOD2 | GXYLT2 | 5.14E-01 | 2.10E-29 | 5.89E-27 |
| PLOD2 | ROR2 | 5.30E-01 | 2.07E-31 | 9.09E-29 |
| PLOD2 | LAMA4 | 5.17E-01 | 9.28E-30 | 2.84E-27 |
| PLOD2 | TRO | 5.10E-01 | 8.60E-29 | 2.26E-26 |
| PLOD2 | GPC6 | 5.33E-01 | 6.91E-32 | 3.73E-29 |
| PLOD2 | EDNRA | 5.26E-01 | 6.47E-31 | 2.47E-28 |
| PLOD2 | C5orf13 | 5.17E-01 | 1.11E-29 | 3.29E-27 |
| PLOD2 | PTGER3 | 5.44E-01 | 2.86E-33 | 2.14E-30 |
| PLOD2 | SGCE | 5.13E-01 | 3.16E-29 | 8.76E-27 |
| PLOD2 | FRMD6 | 5.21E-01 | 3.43E-30 | 1.12E-27 |
| PLOD2 | BEND6 | 5.31E-01 | 1.47E-31 | 6.77E-29 |
| PLOD2 | RASSF8 | 5.45E-01 | 1.70E-33 | 1.37E-30 |
| PLOD2 | PLSCR4 | 5.82E-01 | 5.09E-39 | 1.72E-35 |
| PLOD2 | FERMT2 | 5.32E-01 | 9.87E-32 | 4.95E-29 |
| PLOD2 | PALM2-AKAP2 | 5.28E-01 | 4.09E-31 | 1.62E-28 |
| PLOD2 | RNF217 | 5.02E-01 | 6.48E-28 | 1.54E-25 |
| PLOD2 | DSEL | 5.15E-01 | 1.84E-29 | 5.24E-27 |
| PLOD2 | DZIP1 | 5.43E-01 | 3.30E-33 | 2.38E-30 |
| PLOD2 | MSRB3 | 5.04E-01 | 4.62E-28 | 1.13E-25 |
| PLOD2 | SGIP1 | 5.33E-01 | 7.01E-32 | 3.73E-29 |
| PLOD2 | PHLDB2 | 5.16E-01 | 1.23E-29 | 3.59E-27 |

| | | | | |
|---|---|---|---|---|
| PLOD2 | DPYSL3 | 5.10E-01 | 6.70E-29 | 1.78E-26 |
| PLOD2 | ARMCX2 | 5.03E-01 | 5.19E-28 | 1.25E-25 |
| PLOD2 | GUCY1B3 | 5.13E-01 | 3.49E-29 | 9.41E-27 |
| PLOD2 | RECK | 5.29E-01 | 3.12E-31 | 1.26E-28 |
| PLOD2 | FAM198B | 5.32E-01 | 1.00E-31 | 4.95E-29 |
| PLOD2 | PKD2 | 5.33E-01 | 8.62E-32 | 4.47E-29 |
| PLOD2 | LRCH2 | 5.09E-01 | 8.83E-29 | 2.26E-26 |
| PLOD2 | TRPC1 | 5.32E-01 | 1.05E-31 | 5.08E-29 |
| PLOD2 | CSGALNACT2 | 5.00E-01 | 1.18E-27 | 2.75E-25 |
| PLOD3 | LRWD1 | 6.32E-01 | 9.91E-48 | 6.68E-44 |
| PLOD3 | SLC4A2 | 5.22E-01 | 2.26E-30 | 5.71E-27 |
| PLOD3 | GPR172A | 5.26E-01 | 6.15E-31 | 1.78E-27 |
| PLOD3 | ZNHIT1 | 5.74E-01 | 1.01E-37 | 4.08E-34 |
| PLOD3 | AP1S1 | 6.62E-01 | 1.09E-53 | 1.10E-49 |
| PLOD3 | GNB2 | 5.51E-01 | 2.25E-34 | 7.59E-31 |
| PLOD3 | PTCD1 | 5.12E-01 | 4.48E-29 | 1.01E-25 |
| PLOD3 | SLC12A9 | 5.96E-01 | 2.66E-41 | 1.34E-37 |

[*] Only genes with correlation coefficient > 0.5 are listed.

Supplementary Figure 1

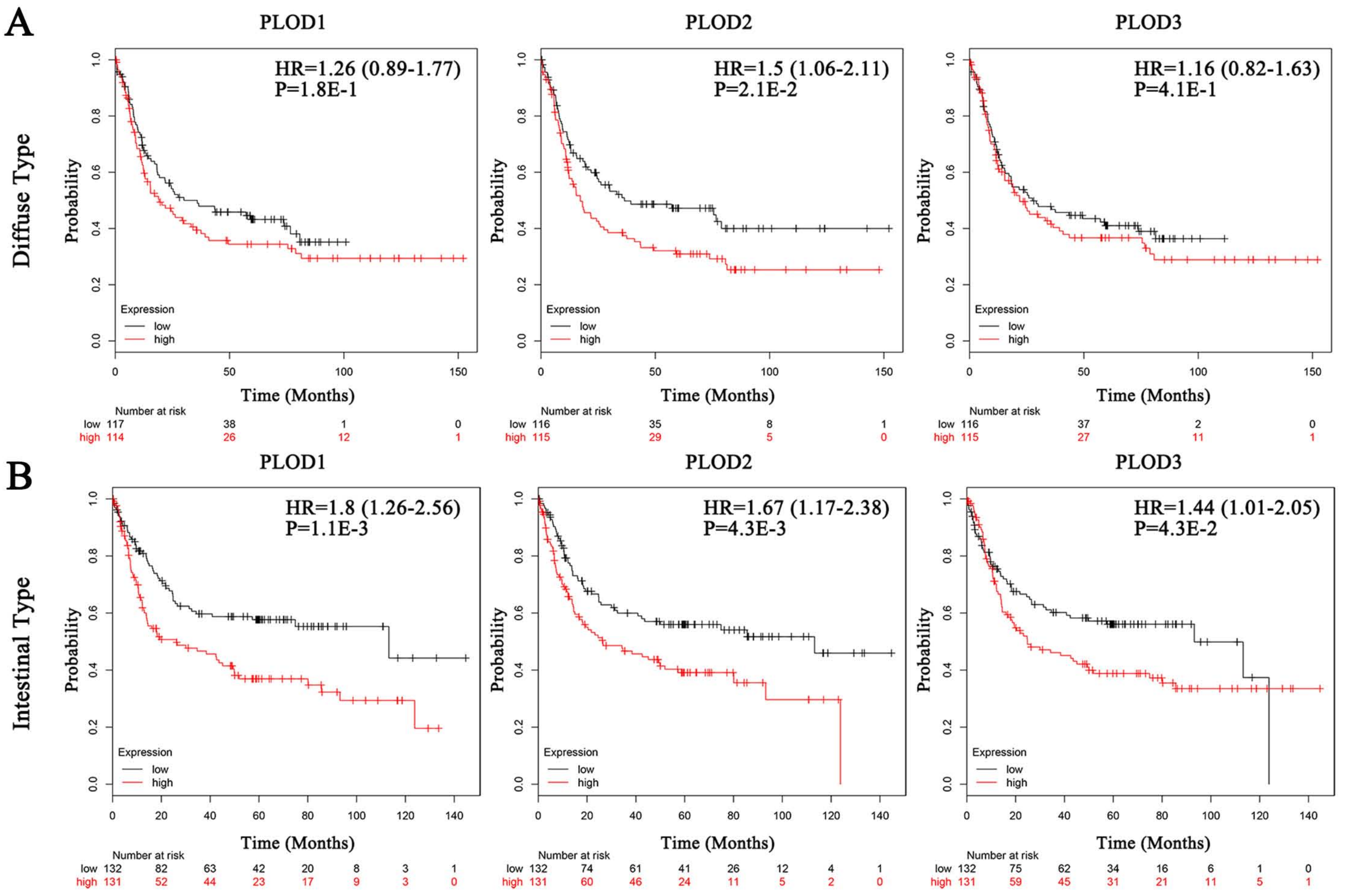

# Supplementary Figure 2

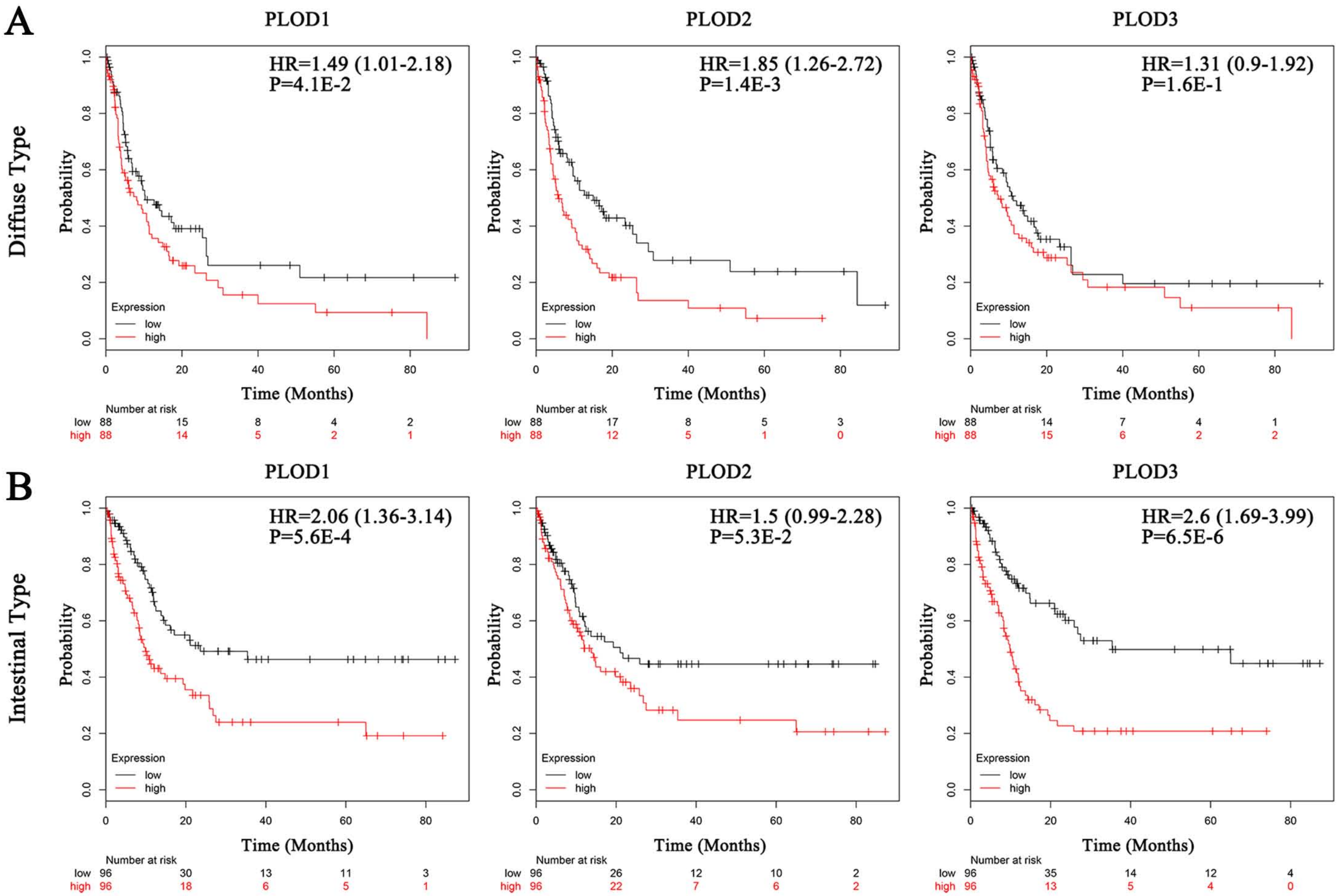

# Supplementary Figure 3

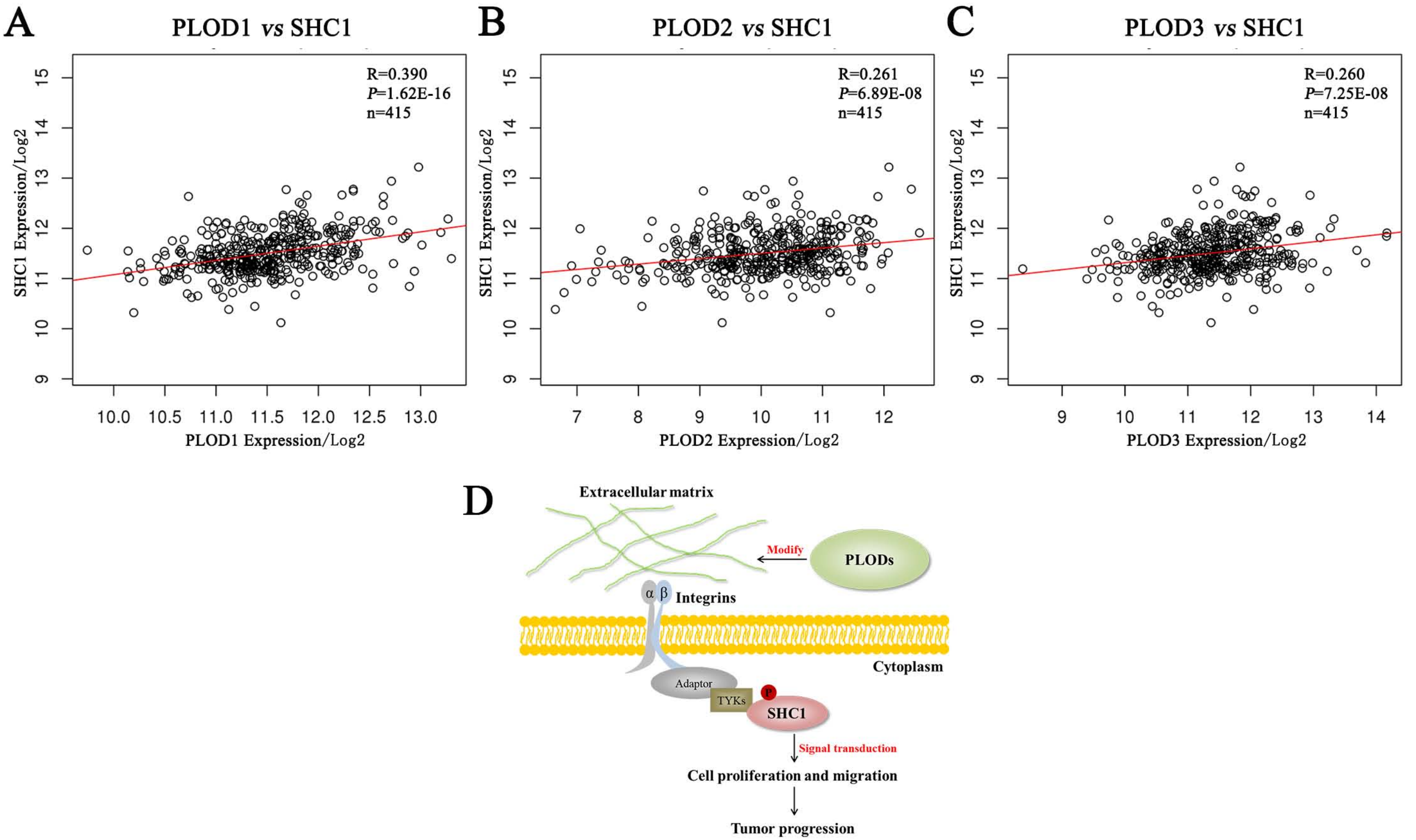

Supplement: Supplementary file 1 — Supplementary figures and tables. [file jcav11p0121s1.pdf]
